# Hands Up, Now What?: Black Families’ Reactions to Racial Socialization Interventions

**DOI:** 10.5195/jyd.2020.755

**Published:** 2020

**Authors:** Riana Elyse Anderson, Isha Metzger, Kimberly Applewhite, Broderick Sawyer, William Jackson, Santos Flores, Amber Majors, Monique Chanel McKenny, Robert Carter

**Affiliations:** University of Michigan; University of Georgia; University of Utah, Utah Center for Evidence Based Treatment; University of Louisville; Village of Wisdom; University of North Carolina, Greensboro; Village of Wisdom; University of Miami; Teachers College, Columbia University

**Keywords:** racial discrimination, racial stress and trauma, coping, racial socialization, interventions

## Abstract

Given the heightened national attention to negative race-related issues and the subsequent community solution-oriented outcry (e.g., Black Lives Matter movement), it is crucial to address healing from racial discrimination for Black Americans. Clinical and community psychologists have responded by developing and implementing programs that focus on racial socialization and psychological wellness, particularly given disproportionate issues with utilization, access, and the provision of quality services within urban and predominantly Black communities. The aim of this article is to describe 2 applied programs (Engaging, Managing, and Bonding through Race and Family Learning Villages), which seek to address and heal racial stress through crucial proximal systems—families and schools—and to highlight participant reactions. These programs offer solutions through strengths-based and participatory approaches which draw from Black Americans’ own protective mechanisms related to improved mental health. We conclude with a discussion on practice, assessments, and models specific to racial stress for researchers, practitioners, and consumers of mental health services.

## Introduction

Over 90% of Black Americans report racially discriminatory experiences over their lifetime (National Public Radio et al., 2017). Unfortunately, a similar proportion of Black American children indicate the same, with the vast majority reporting the encounters in their proximal environments (Pachter et al., 2010). Research has continuously demonstrated the link between racial discrimination and psychological outcomes (Gaylord-Harden & Cunningham, 2009; Nyborg & Curry, 2003; Pieterse et al., 2012). Racial bias in children’s social interactions can occur as early as 3 years of age (Katz, 2003), with discrimination in school environments leading to poorer school outcomes and lower self-esteem over time (Mattison & Aber, 2007).

The need for understanding the impact of racial discrimination has increased in recent years given heightened vicarious racism and violence. The fatal shootings of Black American youth, including Trayvon Martin in 2012 and Michael Brown in 2014, served as a major catalyst for modern-day movements (e.g., Black Lives Matter). Furthermore, mainstream and social media outlets, accelerated through advancing technology, have expanded the reach of graphic race-related traumatic events (Tynes et al., 2008; 2019). The increase in media exposure to race-based violence raises questions about the effect and treatment of vicarious witnessing in Black American communities. This paper investigates the ways in which Black Americans respond to intervention approaches to racial discrimination where racial stress and trauma may be most primed in their proximal environments: the family and school.

## Racial Stress and Trauma

The concept of racial stress and trauma (RST) refers to emotional injury caused by racially motivated stressors that overwhelm one’s coping capacity and impact quality of life or otherwise cause fear, helplessness, and horror, resulting in anxiety, maladaptive coping, negative mental health outcomes, and anger (Bryant-Davis, 2007; Carter, 2007). Clinical and community psychology research indicates that both direct and vicarious exposure to racial discrimination, pre- and post-event (Carter et al., 2013), can cause these trauma-like symptoms (Hoggard et al., 2012; Soto et al., 2011; Terrell et al., 2006). While not every Black American is traumatized by their exposure to racial discrimination, it is crucial through research and treatment to understand how to address negative reactions.

Researchers have challenged traditional notions of trauma, suggesting that the navigation of racial discrimination may be potentially traumatic for minority populations (Holmes et al., 2016). Carter’s (2007) conceptualization of race-based traumatic stress injury (RBTSI) links emotional reactions to a specific encounter with racial discrimination, illuminating the ways in which different dimensions of racism may generate traumatic or other stress responses (Carter & Sant-Barket, 2015). The RBTSI model helps to extend beyond diagnostic categories such as depression, anxiety, post-traumatic stress disorder, and acute stress reactions, particularly given the racial disparities in trauma diagnosis (Himle et al., 2009; Williams et al., 2014).

Unlike other social determinants of health, racialized stressors may be the most challenging for Black Americans to identify and from which to cope (Braveman et al., 2011). Although traditional stress and coping theories identify appraisal as an initial step toward understanding the threat of one’s stressors (Lazarus & Folkman, 1987), RST is often difficult to appraise because both perpetrator and victim live in a culture of incentivized racial avoidance (Carter, 2007; Pinderhughes, 1989; Stevenson, 2014). A primary source of RST for Black American youth is the discrimination they face in school settings (Hope et al., 2015), with up to 93% of Black American youth reporting some form of racial bias in their educational environment (Harris-Britt et al., 2007). As such, discrimination may result in Black American children employing cognitive resources otherwise reserved for learning (e.g., academic curiosity and persistence; Neblett et al., 2006). Given that schools can be a site of racial hostility and families may be a place in which youth naturally go to process the totality of their experiences, it is critical to investigate the viability of both settings as implementation targets for improved race-related practices.

## Racial Socialization

One strategy that caregivers use to address youth’s racial concerns is the practice of racial socialization. Racial socialization, or the verbal and nonverbal messages between caregivers and youth regarding the positive and negative aspects of racialized experiences (Lesane-Brown, 2006), is most frequently utilized as youth reach adolescence, or when awareness of racial biases increases (Hughes et al., 2006). Similar to general coping socialization tasks performed by parents, experiences of racial discrimination may trigger specific coping strategies (e.g., infusing cultural pride [cultural socialization], preparation for bias, generalizing experiences to everyone within that characteristic [promotion of mistrust], and/or not attributing the incident as inherently racist [colorblindness]; R. E. Anderson, Jones, et al., 2018; Hughes et al., 2006). Findings from racial socialization research over the past 40 years generally show that racial socialization constructs are associated with well-being indicators across domains of psychosocial development, identity, and coping (Evans et al., 2012; Gaylord-Harden et al., 2012; Neblett et al., 2009; Seaton et al., 2012). In particular, the Racial Encounter Coping Appraisal and Socialization Theory (RECAST; R. E. Anderson & Stevenson, 2019; Stevenson, 2014) indicates that racial socialization plays a role in the relationship between racial discrimination and youth wellness by enhancing the youth’s self-efficacy and the coping capacity between family members through repeated dialogue and management of stress.

A growing body of research demonstrates that the racial socialization messages Black American youth receive from their parents can protect against the negative effects associated with racial bias (e.g., Neblett et al., 2006). However, Stevenson (2014) has argued that racial coping is not simple, but acquired through knowledge and skill development: “Mostly schools and families fail at teaching racial coping because teachers, administrators, parents, and students are not trained to do so, [and] do not approach it as a competency topic” (pg. 4). Due to a lack of programmatic or curricular instruction in various settings to address racial bias, parents often shoulder the burden of preparing their children to face this stressor themselves. However, few interventions focus on supporting parents in preparing children to cope with RST at home and school or seek to leverage and strengthen existing parent networks to facilitate more intentional peer support.

### The Utility of Racial Socialization in Intervention Programming

Colloquial racial socialization practices, such as “The Talk,” have recently been promoted in print (Hart, 2017), television programs (Barris, 2016), advertisements (Proctor & Gamble, 2017), and documentaries (J. Anderson, 2017). Given the extensive research highlighting the positive impact of racial socialization on the development of Black youth, researchers have called for applied methods and interventions that incorporate this cultural strength (Jones & Neblett, 2016). Existing programs that utilize culturally enhanced practices have generally been implemented within community (e.g., Sisters of Nia; Belgrave et al., 2004), school (e.g., Preventing Long-term Anger and Aggression in Youth; Stevenson, 2003), and family (Black Parenting Strengths and Strategies; Coard et al., 2007; Strong African American Families Program; Murry et al., 2007) systems.

While the psychological field moves towards understanding the basic relationships between racial socialization practices and adolescent coping capacity, little is known about how racial socialization practices can be tailored to reduce both youth and parent RST, particularly through clinical intervention (Coard et al., 2007; Coard et al., 2004). Given heightened relevance of race and racism, particularly after the 2016 U.S. presidential election (Southern Poverty Law Center, 2016), research should continue to explore the ways in which racial socialization can help parents facilitate the active healing process of their children from the effects of past, current, and future discrimination. As such, this article highlights participant responses to two clinical and community-based programs designed to reduce RST through family support and psychoeducation. The two programs discussed address the call for family- and school-based interventions to address RST with racial socialization.

## Section 1: EMBRacing Family-Based Approaches to Combat Racial Discrimination

### Overview

Researchers and practitioners developed the five-session Engaging, Managing, and Bonding through Race (EMBRace) intervention to address the unmet clinical needs of Black American families experiencing RST (R. E. Anderson, McKenny, & Stevenson, 2018). EMBRace encourages the competent development of racial socialization skills for parents and their children to process both individual coping and parent–child dynamics inherent in racial discrimination communication (Dunbar et al., 2017; Smith-Bynum et al., 2016), with the goal of confronting RST as a family. EMBRace has a research-based manualized curriculum that corresponds with the natural use of racial socialization in Black American families (Coard et al., 2004).

### Method

Participants were recruited for the program in a major urban city in the Northeastern United States. Researchers met challenges inherent in community-based recruitment by consulting with focus groups and community partners, and then recruited at community-wide events, through schools, and with snowball sampling. Target youth were 10 to 14-year-olds (*N* = 10, *M*_*age*_ = 13.3, *SD* = 1.09) who identified at least one parent (*N* = 10, *M*_*age*_ = 41.4, *SD* = 7.92) as African American. More than one caretaker was able to participate in the parent sessions if desired. Fifteen families were originally recruited for the study, and 66% (*N* = 10) maintained attendance and enrollment throughout the 7 weeks of the program. Families with more than one child were provided with childcare at the program. Families received a $100 gift card, dinner, and other various in-kind community donations throughout the intervention as incentives. Sessions were provided within the community at neighborhood schools and at an urban-based university setting, in which transportation and/or parking was provided.

Clinicians (counseling, clinical, and community psychologists and social workers) were trained to implement EMBRace. Each facilitator participated in identity exploration prior to sessions to appropriately address the concept of race and racism. Families engaged in a 2-hour pretest measuring racial stress, coping ability, and other well-being constructs via quantitative, qualitative, and observational measures. Throughout the intervention, families were scheduled for 2-hour blocks during the weekday evening hours or weekend afternoons. The sessions had a parallel structure for parents and adolescents during the first 30 minutes. Next, facilitators provided a 15-minute dinner and break to the families. Finally, clinicians met with the family conjointly for 45 minutes. After implementation, families were administered an identical posttest along with items specifically measuring program satisfaction.

The initial four sessions of the program reviewed one component of racial socialization per week (i.e., cultural socialization, preparation for bias, promotion of mistrust, and egalitarianism), with practical application of all components reviewed in session five (R. E. Anderson, McKenny, & Stevenson, 2018). For the present article, the qualitative interviews conducted at posttest were evaluated with the query function in NVivo 11.0 for statements relevant to racialized stress and coping response, especially as it related to the question, “What have you done differently about a racial experience you recently encountered?”.

### Results and Discussion

Participants reported that EMBRace was a safe space to share intimate feelings about their stress. Most parents indicated that EMBRace changed the way they communicated with their child about race and RST. One mother noted, “I have learned so much about myself as a Black woman and raising Black children. EMBRace has helped me talk to my children about being Black in America and understanding how to recognize and deal with any stress being Black may cause.”

The potential intergenerational nature of RST was also emphasized, in which another mother indicated:

Um, I liked that it gave not just [T] but also me the opportunity to talk about race relations, even bringing up some things that I experienced in the past, and you know I didn’t even really know that it was racist or never had the opportunity to talk about. Um, and that it’s okay! Like it’s okay to talk about it. And I think that [T] is sharing what he has learned here with his peers, which is very, very important.

Additionally, EMBRace provided coping strategies to deal with ambiguous RST, as one mother explains:

You don’t really know, so it’s more, it’s more important to learn how to deal with the feelings that you have, um, coming from that negative experience and … and I think that too, like being Black sometimes, like, I don’t know sometimes you just get a gut feeling and you’re like ‘umm … I don’t like the way, you know, that interaction went down’ [laughs] and you kinda have a gut feeling that it was racial but you can’t really prove it and obviously not going to go back to the person and ask them. So again, it’s just focusing on how to deal with those feelings no matter what you perceive the person’s issues were.

Youth participants likewise indicated an improvement in strategies after completing EMBRace, as indicated in this account by an adolescent boy:

Because of EMBRace, I talked to my mom about a time where I was just like standing on a corner and waiting for my mom to pick me up from … practice, and a couple walked by me and they said like “Oh hurry up, I don’t want to get robbed.” And I was the only one there and it was obviously directed at me. The fact is that the instrument on my back was probably far more expensive than anything they were carrying…. So we used some of the EMBRace “funwork” to talk about it and ask each other questions … And it was good to know that someone who had had similar experiences when they were young was also in my corner.

Finally, even for within-group conflict, one adolescent girl noted that EMBRace was useful for her “getting things off [my] chest and getting to the root of why light-skinned and dark-skinned girls don’t get along.” In sum, both parents and youth described elements of EMBRace that helped them address the racialized climate they were facing at the time.

Although incidents of racial discrimination in the United States have been made more widely visible online in the recent decade (Bonilla & Rosa, 2015), families have and will continue to serve as the first line of support for Black American adolescents. Examining how families spoke about racism differently provided insight into behavior shifts over the intervention. Future studies of EMBRace will draw from quantitative and qualitative responses from larger samples to convey findings beyond a trend level. On the whole, however, and given the lack of programming available for families to both explore and cultivate their racial socialization processes, EMBRace participants emphasized its promising contribution to healing from RST for parents and youth through empowerment, culturally relevant content, and a family-focused lens.

## Section 2: It Takes a Village: Building the Community Support Black Families Desire

### Overview

Village of Wisdom (VOW) is a nonprofit organization in an urban Southeastern city designed to enhance Black parent racial socialization and school advocacy and to improve educational attainment and family support. Through VOW, the Family Learning Villages (FLVs) work toward several aims for Black American children, including to: (a) increase teacher expectations, (b) increase school investment in creating more racially affirming events, (c) enhance parent racial socialization strategies, (d) enhance parent advocacy skills, and (e) increase student capacity to envision a future of Black liberation. These programs offer potential solutions through strengths-based and participatory approaches which draw from Black Americans’ own naturalized and protective mechanisms through racial socialization and utilize a framework of reducing RST through evidence-supported coping strategies.

### Method

To recruit families, VOW hosted or co-sponsored large-scale community events that celebrated Black culture, which produced an opportunity to encourage families’ participation in the FLV programming. FLVs consisted of workshops for parent groups facilitated by a team of two trained facilitators that were either contractors or employees of VOW. During FLVs, parents engaged in role play, conversation, and guided reflection on topics including racial bias in school, racial socialization, coping with RST, and discipline.

VOW provided childcare for all children who attended FLVs with their parents. Both individual and coupled parents who participated in FLVs received a $150 gift card, breakfast, and lunch for their participation. Sessions occurred within the community at a local church and school, and transportation was provided to those families who requested it. Parents were also invited to attend FLV “Family Reunions” in which they could connect again with the parents from their particular session and who attended other FLV sessions.

The main goals of FLVs were to increase the number of positive racial socialization messages parents shared with children, improve parent self-efficacy in their ability to advocate on their child’s behalf against school-based RST, and increase the quantity of relationships parents have with other parents. Pre- and post-program surveys were collected during FLVs which yielded both quantitative and qualitative data. Survey items were adapted and taken from various existing instruments. Parents were also given the opportunity to provide short video reflections about their experience in the FLV. Parents were prompted with one question for the video reflections: “What would you tell other parents about the FLV experience?” The data VOW collected was intended to assess the impact of the FLV curriculum on parents and was not conducted through a formal research study.

FLV facilitators utilized data from parents who completed both pretest and posttest measures; thus, 37 parents were involved in the current study. All of the families (100%) had representation from mothers and three (8%) of the families had representation from fathers. Children of the families ranged in age from 6 months to 16 years, but all parents had at least one child above the age of 2 years. Demographic data is not available on the age and number of children within each family.

### Results and Discussion

The total enrollment (*N* = 18) and retention (55.6%; *N* = 10) of the two FLVs provided via a 4-hour, 5-session format was less than desired. Parents cited duration of time and other prior engagements as reasons for not attending all of the 5-session workshop version. Due to the attrition and low enrollment for the 5-session workshops, VOW truncated the structure of the FLV to an 8-hour, 2-day format, leading to higher overall enrollment (*N* = 27) and greater retention (100%; *N* = 27).

Overall, parents reported feeling more efficacious about advocating for their children at school. At pretest, less than half (*n* = 18) of the parents had previously advocated for their children to receive more equitable treatment in schools, however, at posttest, all of the parents (*n* = 37) committed to participate in some form of advocacy (e.g., workshop facilitation, narrative sharing with families and teachers, etc.).

Regarding FLV goals, parents reported an increase in frequency of racial socialization conversations they planned to have with their children. At pretest, 8% (*n* = 3) of parents reported they rarely affirmed their child’s Blackness, 8% (*n* = 3) reported affirming their child’s Blackness once a month, 16% (*n* = 6) reported affirming their child’s Blackness a few times a month, 8% (*n* = 3) reported affirming their child’s Blackness once a week, and 57% (*n* = 22) reported affirming their child’s Blackness a few times a week or almost every day. At posttest, 100% (*n* = 37) of the parents stated that they would affirm their child at least a few times a week or almost every day.

Parent reflections indicated that their participation in FLVs impacted their ability to assist their children in coping with RST. One parent stated “[We discussed] actual racism, discrimination towards children in [our local school system], and how to handle it—react to it and resources that are available that I wasn’t aware of before.” Another parent, reflecting on their plans to facilitate their child’s coping with RST in the future, stated, “[I will] definitely take a moment to breathe so I can address the matter from a centered point. Also call in my support network.” Even more parents reflected on how the FLV facilitated a feeling of community with other parents. “This was a great opportunity to connect with parents who want for their children to have the opportunity to embrace our Blackness in a positive way—and helping to uplift that positivity.” These parent reflections suggest that the FLV experience increased parent awareness of coping strategies that might mitigate racial stress for them and their children in the future.

These initial reactions to VOW’s FLV programming suggest that the organization is potentially creating opportunities for parents to increase the number of racially affirming socialization messages they are sharing with children, learn coping strategies to promote their children’s ability to navigate racial bias, and develop richer relationships with their parent peers to advocate against school-based sources of RST. Future evaluations of the FLV programming should be conducted in a more systematic manner with respect to greater sample sizes and thematic coding and should include longitudinal post-intervention assessments to determine if socialization practices are translating to behavior change. Given the dearth of programming that supports Black parents at the intersection of school advocacy and racial socialization practices, VOW’s FLV workshop series provides a potentially viable model for organizations which seek to enhance parents’ capacity to assist their children in coping with RST.

## Discussion

The authors present parental reactions to healing approaches for RST through family- and school-focused conceptualizations and interventions for Black American youth. The highlighted programs utilize racial socialization as a central mechanism to facilitating positive psychological change and resilience. The EMBRace program is an illustration of how the family is used as an expert-driven therapeutic resource to help heal racial wounds, while the FLVs extend racial socialization practices to the school setting and within the community to promote Black American cultural values and psychological well-being. The sections detailed participant reactions to effective strategies for coping with and processing RST. The reflections presented by both programs indicated that parents benefitted from spaces to discuss racial stress impacting themselves and their children.

The resurfacing of blatant racism in American society has been relatively recent and quite startling in frequency and intensity (e.g., Bassett et al., 2017). Despite the election of the first Black president of the United States leading to hope of a “post-racial America” (Cohen, 2011), continued racial events, especially the highly public deaths of Black American adults and children (Izadi & Holley, 2014), prove otherwise. With the renewed awareness of racism, an opportunity arises for families and schools to address the wounds and emotional and psychological harm of racism and race-based traumatic events.

Limitations of these programs primarily relate to the small sample sizes of the pilot implementation, which limits generalization to other populations. Further, initial measures for both programs were not validated at the time of the implementation. It is important to note, however, that these programs are attempting to fill in gaps from the research, practice, and measurement fields. It has been well established that it takes 17 years for research findings to be translated into practice (Hanney et al., 2015), yet with children and their parents suffering from the effects of RST in the present day, there is a charge to engage in applied methodologies now.

In the development of the RBTSI Model, Carter (2007) indicated that clients who are the targets of racial discrimination could seek a mental health professional for relief from the emotional or psychological effects. In therapy, Black Americans’ perspectives are too often rendered irrelevant and dismissed (Pinderhughes, 1989), with therapists sometimes suggesting that the client’s perceptions are not reality-based (Bonilla-Silva, 2017). Yet objective and subjective appraisals are equally valid as verifiable events when the effect of stress or trauma is in consideration. As such, future studies should better evaluate the impact of these programs not only on the satisfaction of clients to unpack their racial baggage, but also for the impact of clinicians’ growth and training.

To redress concerns of RST, this paper illustrated how burgeoning family and school interventions utilized racial socialization to offer potential solutions for coping with racially discriminatory experiences. Research consistently demonstrates the association between racial discrimination and RST, yet racial socialization offers a potential buffer to these deleterious correlations (R. E. Anderson & Stevenson, 2019). Mental health professionals and other youth-serving agencies (e.g., school personnel) should work to affirm injury resulting from racism in Black American families, examine their own potential racial bias as members of a racially charged society, and treat racist experiences as occurrences which warrant redress. As such, the translational programs from this article can serve as an initial consideration for mental health professionals’ understanding, empowerment, and treatment of RST for Black American families.

## References

[R1] Anderson J (Executive Producer). (2017). The talk – Race in America. [TV Special]. The Corporation for Public Broadcasting.

[R2] Anderson RE, Jones S, Anyiwo N, McKenny M, & Gaylord-Harden NK (2018). What’s race got to do with it? Racial socialization’s contribution to black adolescent coping. Journal of Research on Adolescence 29(4), 822–831. 10.1111/jora.12440 30099797 PMC8807351

[R3] Anderson RE, McKenny MC, & Stevenson HC (2018). EMBRace: Developing a racial socialization intervention to reduce racial stress and enhance racial coping among black parents and adolescents. Family Process, 58(1), 53–67. 10.1111/famp.12412 30552778 PMC8807350

[R4] Anderson RE, & Stevenson HC (2019). RECASTing racial stress and trauma: Theorizing the healing potential of racial socialization in families. American Psychologist, 74(1), 63–75. 10.1037/amp0000392 30652900 PMC8807344

[R5] Barris K (Writer), & McCarthy-Miller B (Director). (2016, February 24). Hope (Season 2, Episode 26) [TV series episode]. In Anderson A, Barris K, Dobbins EB, Fishburne L, & Groff J (Executive Producers), Black-ish. Khalabo Ink Society; Wilmore Films; Artists First; Cinema Gypsy Productions; ABC Studios.

[R6] Bassett MT, Krieger N, & Bailey Z (2017). Charlottesville: blatant racism, not grievances, on display. The Lancet, 390(10109), 2243. 10.1016/S0140-6736(17)32855-6 29165264

[R7] Belgrave FZ, Reed MC, Plybon LE, & Corneille M (2004). The impact of a culturally enhanced drug prevention program on drug and alcohol refusal efficacy among urban African American girls. Journal of Drug Education, 34(3), 267–279. 10.2190/H40Y-D098-GCFA-EL74 15648887

[R8] Bonilla Y & Rosa J (2015). #Ferguson: Digital protest, hashtag ethnography, and the racial politics of social media in the United States. American Ethnologist, 42, 4–17. 10.1111/amet.12112

[R9] Bonilla-Silva E (2017). Racism with racists: Color Blind Racism and the persistence of racial inequality in America (5^th^ ed.). Rowman & Littlefield.

[R10] Braveman P, Egerter S, & Williams DR (2011). The social determinants of health: Coming of age. Annual Review of Public Health, 32, 381–98. 10.1146/annurev-publhealth-031210-101218 21091195

[R11] Bryant-Davis T (2007). Healing requires recognition: The case for race-based traumatic stress. Counseling Psychologist, 35, 135–143. 10.1177/0011000006295152

[R12] Carter RT (2007). Racism and psychological and emotional injury: Recognizing and assessing race-based traumatic stress. The Counseling Psychologist, 35, 13–105. 10.1177/0011000006292033

[R13] Carter RT, Mazzula S, Victoria R, Vazquez R, Hall S, Smith S, Sant-Barket S, Forsyth J, Bazelais K, & Williams B (2013). Initial development of the Race-Based Traumatic Stress Symptom Scale: Assessing the emotional impact of racism. Psychological Trauma, 5, 1, 1–9. 10.1037/a0025911

[R14] Carter RT, & Sant-Barket SM (2015). Assessment of the impact of racial discrimination and racism: How to use the Race-Based Traumatic Stress Symptom Scale in practice. Traumatology, 21(1), 32–39. 10.1037/trm0000018

[R15] Coard SI, Foy-Watson S, Zimmer C, & Wallace A (2007). Considering culturally relevant parenting practices in intervention development and adaptation: A randomized controlled trial of the black parenting strengths and strategies (BPSS) program. The Counseling Psychologist, 35, 797–820. 10.1177/0011000007304592

[R16] Coard SI, Wallace SA, Stevenson HC, & Brotman LM (2004). Towards culturally relevant preventive interventions: The consideration of racial socialization in parent training with African American families. Journal of Child and Family Studies, 13, 277–293. 10.1023/B:JCFS.0000022035.07171.f8

[R17] Cohen CJ (2011). Millennials & the myth of the post-racial society: Black youth, intra-generational divisions & the continuing racial divide in American politics. Daedalus, 140, 197–205. 10.1162/DAED_a_00087

[R18] Dunbar AS, Leerkes EM, Coard SI, Supple AJ, & Calkins S (2017). An integrative conceptual model of parental racial/ethnic and emotion socialization and links to children’s social-emotional development among African American families. Child Development Perspectives, 11(1), 16–22. 10.1111/cdep.12218

[R19] Evans AB, Banerjee M, Meyer R, Aldana A, Foust M, Rowley S (2012). Racial socialization as a mechanism for positive development among African American youth. Child Development Perspectives, 6, 2512013257. 10.1111/j.1750-8606.2011.00226.x

[R20] Gaylord-Harden NK, Burrow AL, & Cunningham JA (2012). A cultural-asset framework for investigating successful adaptation to stress in African American youth. Child Development Perspectives, 6, 264–271. 10.1111/j.1750-8606.2012.00236.x

[R21] Gaylord-Harden NK & Cunningham JA (2009). The impact of racial discrimination and coping strategies on internalizing symptoms in African American youth. Journal of Youth and Adolescence, 38, 532–43. 10.1007/s10964-008-9377-5 19636726

[R22] Hanney SR, Castle-Clarke S, Grant J, Guthrie S, Henshall C, Mestre-Ferrandiz J, Pistollato M, Pollitt A Sussex J, & Wooding S (2015). How long does biomedical research take? Studying the time taken between biomedical and health research and its translation into products, policy, and practice. Health research policy and systems, 13, 1. 10.1186/1478-4505-13-1 25552353 PMC4297458

[R23] Harris-Britt A Valrie CR, Kurtz-Costes B, & Rowley SJ (2007). Perceived racial discrimination and self-esteem in African American youth: Racial socialization as a protective factor. Journal of Research on Adolescence, 17, 669–682. 10.1111/j.1532-7795.2007.00540.x 20700391 PMC2917995

[R24] Hart B (2017, May 4). Giving black children ‘the talk’ won’t save them from police brutality. Online. Retrieved February 1, 2018, from https://www.huffingtonpost.com/entry/its-time-to-rethink-the-talk-teaching-black-kids_us_590a133ee4b084f59b49fef0.

[R25] Himle JA, Baser RE, Taylor RJ, Campbell RD & Jackson JS (2009). Anxiety disorders among African Americans, Blacks of Caribbean descent, and non-Hispanic Whites in the United States. Journal of Anxiety Disorders, 2009, 23, 578–590. 10.1016/j.janxdis.2009.01.002 19231131 PMC4187248

[R26] Hoggard LS, Byrd CM, & Sellers RM (2012). Comparison of African American college students’ coping with racially and nonracially stressful events. Cultural Diversity and Ethnic Minority Psychology, 18, 329–339. 10.1037/a0029437 22866688

[R27] Holmes SC, Facemire VC, & DaFonseca AM (2016). Expanding criterion A for posttraumatic stress disorder: Considering the deleterious impact of oppression. Traumatology, 22, 314–321. 10.1037/trm0000104

[R28] Hope EC, Skoog AB, & Jagers RJ (2015). “It’ll never be the white kids, it’ll always be us”: Black high school students’ evolving critical analysis of racial discrimination and inequity in schools. Journal of Adolescent Research, 30, 83–112. 10.1177/0743558414550688

[R29] Hughes D, Rodriguez J, Smith EP, Johnson DJ, Stevenson HC, & Spicer P (2006). Parents’ ethnic-racial socialization practices: a review of research and directions for future study. Developmental psychology, 42, 747. 16953684 10.1037/0012-1649.42.5.747

[R30] Izadi E & Holley P (2014, November 26). Video shows Cleveland officer shooting 12-year-old Tamir Rice within seconds, The Washington Post.

[R31] Jones SCT & Neblett E (2016). Racial-ethnic protective factors and mechanisms in psychosocial prevention and intervention programs for black youth. Clinical Child and Family Psychology Review, 19, 134–161. 10.1007/s10567-016-0201-6 27083688

[R32] Katz PA (2003). Racists or tolerant multiculturalists? How do they begin?. American Psychologist,58 (11), 897–909. 14609382 10.1037/0003-066X.58.11.897b

[R33] Lazarus RS, & Folkman S (1987). Transactional theory and research on emotions and coping. European Journal of personality, 1, 141–169. 10.1002/per.2410010304

[R34] Lesane-Brown CL (2006). A review of race socialization within black families. Developmental Review, 26(4), 400–426. 10.1016/j.dr.2006.02.001

[R35] Mattison E, & Aber MS (2007). Closing the achievement gap: The association of racial climate with achievement and behavioral outcomes. American Journal of Community Psychology, 40, 1–12. 10.1007/s10464-007-9128-x 17587175

[R36] MurryVM, BerkelC, BrodyGH, GibbonsM, & GibbonsFX (2007). The Strong African American Families program: Longitudinal pathways to sexual risk reduction. Journal of Adolescent Health, 41, 333–342. 10.1016/j.jadohealth.2007.04.00317875458

[R37] National Public Radio, Robert Wood Johnson Foundation, & Harvard University T.H. Chan School of Public Health. (2017). Discrimination in America: Experiences and views of African Americans. Robert Wood Johnson Foundation.

[R38] Neblett EW, Philip CL, Cogburn CD, & Sellers RM (2006). African American adolescents’ discrimination experiences and academic achievement: Racial socialization as a cultural compensatory and protective factor. Journal of Black Psychology, 32, 199–218. 10.1177/0095798406287072

[R39] Neblett EW, Smalls CP, Ford KR, Nguyen HX & Sellers RM (2009). Racial socialization and racial identity: African American parents’ messages about race as precursors to identity. Journal of Youth and Adolescence, 38, 189–203. 10.1007/s10964-008-9359-7 19636717

[R40] Nyborg VM & Curry JF (2003). The impact of perceived racism: Psychological symptoms among African American boys. Journal of Clinical Child & Adolescent Psychology, 32, 258–266. 10.1207/S15374424JCCP3202_11 12679284

[R41] Pachter LM, Bernstein BA, Szalacha LA, & Garcia Coll C (2010). Perceived racism and discrimination in children and youths: An exploratory study. Health and Social Work, 35, 61–69. 10.1093/hsw/35.1.61 20218454

[R42] Pinderhughes E (1989). Understanding race, ethnicity, and power: The key to efficacy in clinical practice. Simon and Schuster.

[R43] Pieterse AL, Todd NR, Neville HA, & Carter RT (2012). Perceived racism and mental health among black American adults: A meta-analytic review. Journal of Counseling Psychology, 59, 1–9. 10.1037/a0026208 22059427

[R44] Proctor & Gamble. (2017). The Talk [Advertisement]. Retrieved February 1, 2018 from https://www.pgeveryday.com/tag/mbib-the-talk

[R45] Seaton EK, Yip T, Morgan-Lopez A, Sellers RM (2012). Racial discrimination and racial socialization as predictors of African American adolescents’ racial identity development using latent transition analysis. Developmental Psychology, 48, 448–458. 10.1037/a0025328 21875184 PMC3748594

[R46] Smith-Bynum MA, Anderson RE, Davis BL, Franco MG, & English D (2016). Observed racial socialization and maternal positive emotions in African American mother–adolescent discussions about racial discrimination. Child development, 87, 1926–1939. 10.1111/cdev.12562 27211821 PMC5121096

[R47] Soto JA, Dawson-Andoh NA, & BeLue R (2011). The relationship between perceived discrimination and generalized anxiety disorder among African Americans, Afro Caribbeans and Non-Hispanic Whites. Journal of Anxiety Disorders, 25, 258–265. 10.1016/j.janxdis.2010.09.011 21041059 PMC3053120

[R48] Southern Poverty Law Center. (2016). Update: 1,094 bias-related incidents in the month following the election. Southern Poverty Law Center: Hatewatch. https://www.splcenter.org/hatewatch/2016/12/16/update-1094-bias-related-incidents-month-following-election

[R49] Stevenson HC (2003). Playing with anger: Teaching coping skills to African American boys through athletics and culture. Greenwood Publishing Group.

[R50] Stevenson HC (2014). Promoting racial literacy in schools: Differences that make a differenc. Teachers College Press.

[R51] Terrell F, Miller AR, Foster K, & Watkins CE Jr (2006). Racial discrimination-induced anger and alcohol use among black adolescents. Adolescence, 41(163), 485–92. 17225663

[R52] Tynes BM, Giang MT, Williams DR, & Thompson GN (2008). Online racial discrimination and psychological adjustment among adolescents. Journal of Adolescent Health, 43, 565–569. 10.1016/j.jadohealth.2008.08.021 19027644

[R53] Williams MT, Malcoun E, Sawyer B, Davis DM, Bahojb-Nouri LV, & Leavell Bruce S (2014). Cultural adaptations of prolonged exposure therapy for treatment and prevention of posttraumatic stress disorder in African Americans. Behavioral Sciences, 4, 102–124. 10.3390/bs4020102 25379272 PMC4219246

